# Public Swimming Baths

**Published:** 1897-04-17

**Authors:** 


### PUBLIC SWIMMING BATHS.

"A Swimmer" writes: In reference to your recent article on the pollution of the water in public swimming baths, the evil undoubtedly exists, and it is probably a serious one where the baths are used largely by the inhabitants of poor neighbourhoods. No one, however, who has seen the juvenile portion of these inhabitants enjoying the delights of swimming and splashing about in a large bath would like to deprive them or their elders of this most valuable form of recreation, and the right remedy would seem to be to insist, at all public baths, that every visitor should take a douche bath before he enjoys his swim. I believe this practice is followed at some continental baths, if not in this country, and there are special appliances constructed for providing a rapid wash with the minimum waste of water.

